# Health Risks Awareness of Electronic Waste Workers in the Informal Sector in Nigeria

**DOI:** 10.3390/ijerph14080911

**Published:** 2017-08-13

**Authors:** Chimere M. Ohajinwa, Peter M. Van Bodegom, Martina G. Vijver, Willie J. G. M. Peijnenburg

**Affiliations:** 1 Institute of Environmental Sciences (CML), Leiden University, P.O. Box 9518, 2300 RA, 2311 EZ Leiden, The Netherlands; p.m.van.bodegom@cml.leidenuniv.nl (P.M.V.B.); vijver@cml.leidenuniv.nl (M.G.V.); peijnenburg@cml.leidenuniv.nl (W.J.G.M.P.); 2 Center for Safety of Substances and Products, National Institute of Public Health and the Environment (RIVM), P.O. Box 1, 3720 BA Bilthoven, The Netherlands

**Keywords:** electronic waste workers, health risk knowledge, attitude, practice, informal sector, informal economy, Nigeria

## Abstract

Insight into the health risk awareness levels of e-waste workers is important as it may offer opportunities for better e-waste recycling management strategies to reduce the health effects of informal e-waste recycling. Therefore, this study assessed the knowledge, attitude, and practices associated with occupational health risk awareness of e-waste workers compared with a control group (butchers) in the informal sector in Nigeria. A cross-sectional study was used to assess health risk awareness of 279 e-waste workers (repairers and dismantlers) and 221 butchers from the informal sector in three locations in Nigeria in 2015. A questionnaire was used to obtain information on socio-demographic backgrounds, occupational history, knowledge, attitude, and work practices. The data was analysed using Analysis of Variance. The three job designations had significantly different knowledge, attitude, and practice mean scores (*p* = 0.000), with butchers consistently having the highest mean scores. Only 43% of e-waste workers could mention one or more Personal Protective Equipment needed for their job compared with 70% of the butchers. The health risk awareness level of the e-waste workers was significantly lower compared with their counterparts in the same informal sector. A positive correlation existed between the workers’ knowledge and their attitude and practice. Therefore, increasing the workers’ knowledge may decrease risky practices.

## 1. Introduction

Across the world, electronic or electrical devices have become indispensable in our daily lives, which has led to an exponential demand for electronic equipment and a rapid increase in the rate of electronic waste (e-waste) generation [1]. E-waste, also known as Waste Electrical and Electronic Equipment (WEEE), consists of electrical and electronic devices including all separate components such as batteries at the end of their useful life [1,2,3,4]. E-waste is one of the fastest growing municipal waste streams. The annual growth rate is 3–5%, which is approximately three times faster than other municipal solid waste. Globally, 50 million metric tons of e-waste is estimated to be generated in 2018 [1]. E-waste contains over 1000 different substances, some of which are hazardous substances (such as lead, mercury, cadmium, arsenic and beryllium) and persistent organic pollutants (including polychlorinated biphenyls and brominated flame retardants) [5]. About 80% of the e-waste generated globally is recycled in informal settings in developing countries such as Nigeria, Ghana, China and India [6].

Informal work is defined as all economic activities by workers and economic units that are in law or in practice not or insufficiently covered by formal arrangements, i.e., beyond the reach of formal laws [7]. Workers are casually employed, often by family members or are self-employed and do not have job security or benefit from social protection [8]. In addition many of them are not aware of available protections or their legal rights. In developing countries, the informal sector is vast and cuts across several different economic fields, including e-waste recycling. It provides services at low cost and is characterised by unsafe working conditions and poor health standards [9,10,11]. Informal economy flourishes in a context of soaring unemployment (formal). As a fast growing sector, informal work provides employment for the majority of the African and Asian populations, covering 66% of employment in Sub-Saharan Africa and 82% in South Asia excluding employment in the agricultural sector [10]. Most of the workers work mainly for economic benefits. In this sector, labour standards are not enforced, lack of regulations prevails, official governance is lacking, and the sector is generally overlooked [10,11]. In Africa, over 300,000 work-related deaths, over 44,000 work-related injuries occurred, and over 49 million workers were absent from work for at least four days due to occupational accidents in 2008 [12,13]. Globally, an estimated 2.3 million work-related deaths occur annually, and the economic cost of work-related injury and illness is estimated at 4% of the world’s GDP (Gross Domestic Product) [12,13]. Furthermore, about 2.9 billion workers globally are estimated to be at risk at work, and about 3.5 years of healthy life are lost per 1000 workers globally. Figures of work-related injuries and deaths in the informal sector alone are unavailable. E-waste recycling industry is a young rapidly growing industry. It has created many employment opportunities; affordable access to electronics and parts used for repairs; a continuous supply of raw materials to manufacturers without further exploration of natural resources; and conservation of natural resources and energy required to manufacture new electronics from virgin resources [14]. Informal recycling involves labor-intensive manual dismantling, isolation of materials, open burning of plastics from electronics, heating of circuit boards, use of toxic acid baths for metal recovery as practiced in Asia, and open dump disposal [15,16]. These unsafe recycling techniques are used to recover valuable materials without or with very little technology to minimise exposure, thus allowing the emission of dangerous chemicals. Occupational safety and environmental protection are clearly not prioritised.

Studies have shown that exposure to the mixture of chemicals emitted during e-waste processing is harmful to human health and induces adverse effects including skin disease, under-development of the brain in children [17], damage to the nervous system, malfunctioning of the kidneys, respiratory problems, endocrine disruption, adverse pregnancy and birth outcomes, and poor health burden heritage perpetuated through the mother-to-child etc. [2,18,19]. These health problems are most evident when there is direct occupational exposure like in the case of informal e-waste workers. Despite the risks associated with informal e-waste recycling, there is generally low awareness of the environmental and health risks associated with informal recycling of e-waste, even among e-waste workers themselves [2,15,19,20]. There is a need for the e-waste workers to understand the integral link between their occupation and their health. As a result of international attention to the negative impacts of e-waste activities on the environment and human health [21]. Nigeria is a signatory to international treaties (such as the Basel convention) [22] and has national legislations regulating import and management of e-waste in Nigeria [23,24]. Unfortunately, the enforcement of these legislations are weak.

In 2014, Nigeria generated about 219 kilo tonnes of e-waste [1], which is largely recycled by the informal sector [25]. In 2005, an estimated 60,000 metric tonnes of used or scrap computers were imported and about 25–75% of these were non-functional [26,27]. There is limited attention and information on informal e-waste workers’ awareness of the risks inherent in unsafe recycling of e-waste and their work conditions in Nigeria. This poses a challenge to policy makers to design effectively environmentally sound e-waste management strategies, or tailor made intervention programmes for the reduction/prevention of the negative health effects of informal e-waste recycling.

If the level of knowledge correlates with practice and attitude, increasing knowledge of the workers may decrease risky practices; suggesting that a bottom-up approach in tackling the negative effects of e-waste recycling may be an effective strategy to improve the workers’ condition. Considering the health risks of exposure to e-waste chemicals, this study therefore aims to answer the following questions:

- To what extent are informal e-waste workers in Nigeria aware of health risks inherent in their daily jobs?
- Are workers in other job types (job designations) in the informal sector more aware of their occupational health risks compared with e-waste workers?
- What factors influence their awareness level across various job types?
- What factors contribute to the difference in the awareness level across various job designations?

In this study, awareness was assessed by measuring the workers’ knowledge, attitude and practices (KAP) on health risks inherent in their daily jobs. We assessed awareness level by using dedicated KAP questions.

## 2. Methods

### 2.1. Study Location

**Figure 1:**
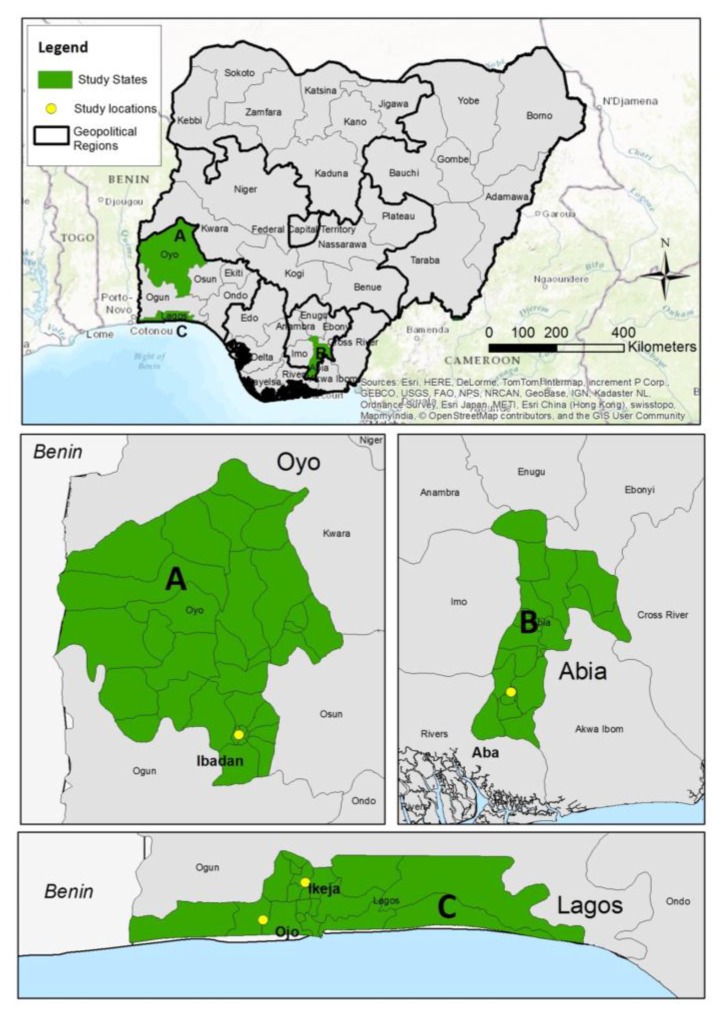
Map of Nigeria showing the study locations [28].

The study was conducted in three study locations/cities (Ibadan, Lagos, and Aba) in Nigeria. The three locations are among the large cities where e-waste is recycled. Ibadan (in Oyo State) and Lagos are located in the South-West and Aba (in Abia State) is located in the South-East geopolitical zones of the country [25]. Figure 1 presents a map of Nigeria showing the study locations. 

### 2.2. Study Design

A comparative cross-sectional study design was adopted to gain insight into the awareness level of e-waste workers compared with a similar occupational group in the informal sector in Nigeria. For this study we selected butchers as a control group for the comparison in each study location, because like e-waste workers, butchers constitute a male dominated profession of a comparable socio-economic status in the informal sector, although, a small part of the meat sector is regulated by the government, it is largely an informal sector. Moreover, butchers are also engaged in small-scale enterprises. They make a living from slaughtering and selling meat in abattoirs and markets within the city. Their work involves contact with live animals, their carcasses, blood and body fluids. The e-waste workers were split into two job designations (repairers and dismantlers). The repairers repair or refurbish electronics, while the dismantlers collect/scavenge, dismantle, and burn e-waste to recover valuable materials.

**Figure 2:**
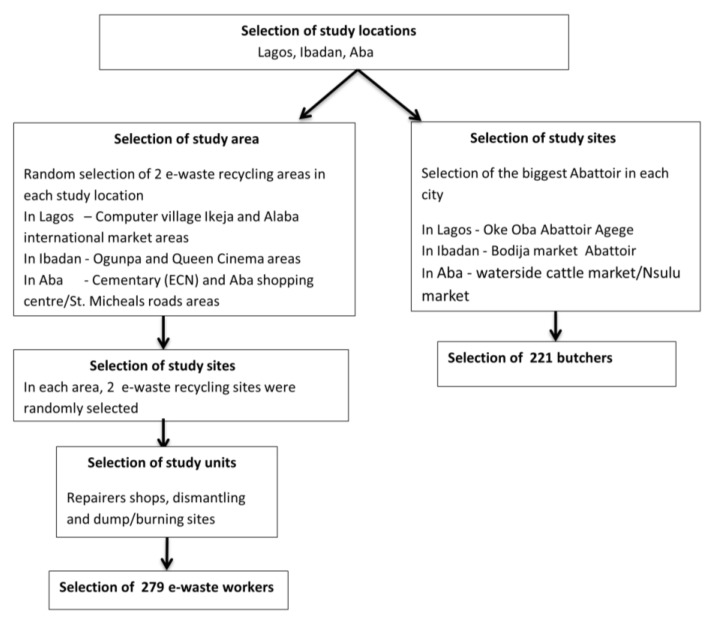
Flow diagram for sample selection.

A multi-stage sampling technique was used to select a minimum of 74 e-waste workers and 74 butchers from each study location. The minimum sample size calculated for both groups was 444 (including adjustment for 10% non-response rate). At each study location, there were a couple of areas where e-waste is recycled, of which two major e-waste recycling areas were selected. For Lagos the areas included the computer village Ikeja and the Alaba international market. In Aba, the shopping centre and Cemetery and Jubilee road/St Michael’s road areas were selected, while in Ibadan the Ogunpa and Queens Cinema areas were randomly selected. In each selected study area, two sampling sites were randomly selected in order to ensure sufficient samples for the required sample size. Each sampling site comprised of hundreds of units/clusters/shops where e-waste is either repaired/refurbished or dismantled/recycled. At each sampling site, systematic sampling was used to select the recycling units. The participants were selected from the recycling units to ensure that the selected participants are a representative sample of the selected area. Regarding the butchers, they were selected from Oke Oba Abattoir Agege in Lagos, Bodija market in Ibadan, and in Aba, Waterside cattle/Nsulu markets were selected. Figure 2 presents a schematic diagram of the sample selection.

### 2.3. Data Collection

A semi-structured interviewer-administered questionnaire containing open- and closed-ended questions (see Supplementary Materials) was used to obtain information from the respondents between May and October 2015. One week prior to the actual data collection period, the questionnaire was pre-tested at an area different from the selected sampling areas and the questions were modified based on the experiences gained during the pre-test. Daily monitoring and evaluation was carried out to ensure accurate data collection. The workers were interviewed on their socio-demographic backgrounds, occupational history, knowledge, attitude, and work practices (KAP) to assess their awareness level.

### 2.4. Data Analysis

Prior to data analysis, all questionnaires were reviewed for completion and accuracy and compiled in a database. The workers’ knowledge on potential health risk as a result of their jobs was assessed by using five questions regarding knowledge of e-waste chemicals, their effects on health, transmission routes, likely health problems as a result of the job, and Personal Protective Equipment (PPE) needed for their jobs. For this study, gloves, work shoes/boots, nose masks (or cloth handkerchiefs improvised as nose mask), any form of head cover, safety glasses/eye protection, ear plugs and coverall/protective work clothes were considered as PPE. Similarly, attitude related to health risks at work was assessed using three questions concerning the workers’ perception of injuries at work, sicknesses that resulted in absence from work, perception (i.e., concerns about work) of the workers of their health, and their major challenges and concern while at work. Safety practices were assessed using five questions on the use of PPE, laundering of PPE at home, washing hands before eating while at work, change of work clothes after work, shower after the day’s work before going home, and carrying heavy loads at work. Each right and wrong/I don’t know response was given a score of 1 and 0, respectively for close-ended questions, but a maximum score of 2 was given for correct responses to open-ended (unprompted) questions. The scores were converted to a 1-to-10-point scale. 

To evaluate the differences in the mean knowledge, attitudes and practices scores between job designations, a series of one-way ANOVA (for categorical variables) and linear regressions (for continuous variables) were run after checking that skewness and kurtosis satisfied the assumption of normality with values less than I2.0I and I9.0I respectively [29]. The explanatory variables tested were job designation, location, migration status, education, worker’s position in the business, use of PPE, age, and number of years of work experience. The factors that were significant and not strongly collinear to other explanatory variable were selected for further analysis. To get a deeper understanding of the nature of the significant differences, series of two-way ANOVA were run always with job designation as the first factor (given the prime interest in the effects of e-waste workers vs. butchers) and the additional selected variables as second factor. Bonferroni post-hoc tests were included to interpret the significant main effects. All analyses were performed using SPSS version 23.0. In all cases, a *p*-value < 0.05 was considered to be statistically significant.

### 2.5. Ethical Considerations

Ethical approval was obtained from the University of Ibadan/University College Hospital Ethical Review Board (No. UI/EC/15/0096). Verbal and written consent of the workers was obtained at the start of the interview, after explaining to the workers their full rights to refuse and to withdraw at any time during the interview. To ensure that the participant remains anonymous each questionnaire was coded with number identifiers. They were also assured that the data will not be used for other purposes than for scientific reasons and for the development of safety promotion programs for the sector. Permission to conduct the study was also obtained from the butchers’ union at each abattoir/market and from association of second-hand electronics dealers at each study site.

## 3. Results

### 3.1. Socio-Demographic and Occupational Characteristics of the Respondents

A total of 279 e-waste workers (55% repairers and 45% dismantlers) and 221 butchers were interviewed. The e-waste workers were younger with a mean age of 30 ± 9 years (repairers 32 ± 8, and dismantlers 29 ± 9) compared to the butchers with a mean age of 40 ± 11 years. Years of work experience ranged from 1 to 32 years for e-waste workers and 1–45 years for butchers. Most of them (98% of the e-waste workers and 82% of the butchers) worked six days a week, and the mean number of working hours per day was 9 ± 2 h for e-waste workers, and 9 ± 3 h for the butchers. A majority (89% of e-waste workers and 93% of the butchers) of the respondents were permanent workers in their profession. Before starting the work, 81% of the e-waste workers and 76% of butchers had at least some form of training, although most (98% of e-waste workers and 99.5% of butchers) of the training was on-the-job training and 88% of e-waste workers and 100% of the butchers got all their training from their employer/senior apprentice. The descriptive statistics associated with the knowledge, attitude, and practice scores of the participants across job designations and other variables are reported in Table 1.

### 3.2. Assessment of Knowledge on Occupational Health Risk

The majority (88%) of e-waste workers (repairers 95%, dismantlers 79%) were unable to mention at least one chemical present in e-waste, and were unaware that e-waste contains hazardous chemicals which could harm their health, while none (0%) of the butchers was unaware that the materials from the animals could pose a health risk. Only 43% of the e-waste workers (37% repairers and 50% dismantlers) and 59% of butchers could mention at least one route of exposure. Only 43% of e-waste workers (34% repairers, 53% dismantlers) and 70% of butchers could mention at least one PPE needed for their job. The majority (77%) of the e-waste workers (82% repairers and 76% dismantlers) and 74% of the butchers did not know the likely illnesses they can contract as a result of their jobs. Overall 70% of the e-waste workers and 78% of the butchers did not think that the substances they are exposed to at work can pose any health risk. In addition, they did not think that they can get ill from their jobs, but from other sources unrelated to their work or work environment. Overall, only 12% of the e-waste workers (repairers 3%, dismantlers 24%) compared with 76% of the butchers had a total score of >5 and were categorized as having good knowledge of their occupational health risk. The mean knowledge scores of the different job designations were repairers 2.6 ± 1.2, dismantlers 3.7 ± 1.8, and butchers 5.8 ± 1.2, reflecting that the butchers had a much better knowledge about the health risks their jobs poses. The mean knowledge scores were significantly different (F (2497) = 269.582, *p* = 0.000, ŋ^2^ = 0.520; indicating that 52% of the variation in knowledge score was explained by job designation) (see Table 1 and Table 2). There was more variation among the dismantlers compared to the other work groups with repairers showing the least variability.

### 3.3. Factors that Influence the Knowledge Scores of the Workers

The multicollinearity analysis revealed a weak correlation between job designation and ethnicity, migration status, position in business, and use of Personal Protective Equipment (PPE); also, there was a weak correlation between age and years of work experience. However, a strong correlation was found between job designation and education, the repairers were more educated than the other groups. In addition, there was a strong correlation between location and ethnicity; each location had a particular dominant ethnic group.

To understand the factors that could influence the scores across the job designations (which is the prime interest), a series of one-way ANOVA (for categorical variables) and linear regressions (for continuous variables) were run. Job designation might contribute to a worker’s awareness level of occupational health risk, but that effect may differ across locations, educational status, age, job position in a business and use of PPE (see Table 2). To evaluate the nature of the significant differences in the mean scores, series of two-way ANOVA were run always with job designation as the first factor (given the prime interest in the effects of e-waste workers vs. butchers) and the additional selected explanatory (predicted) variables as second factor. 

**Figure 3:**
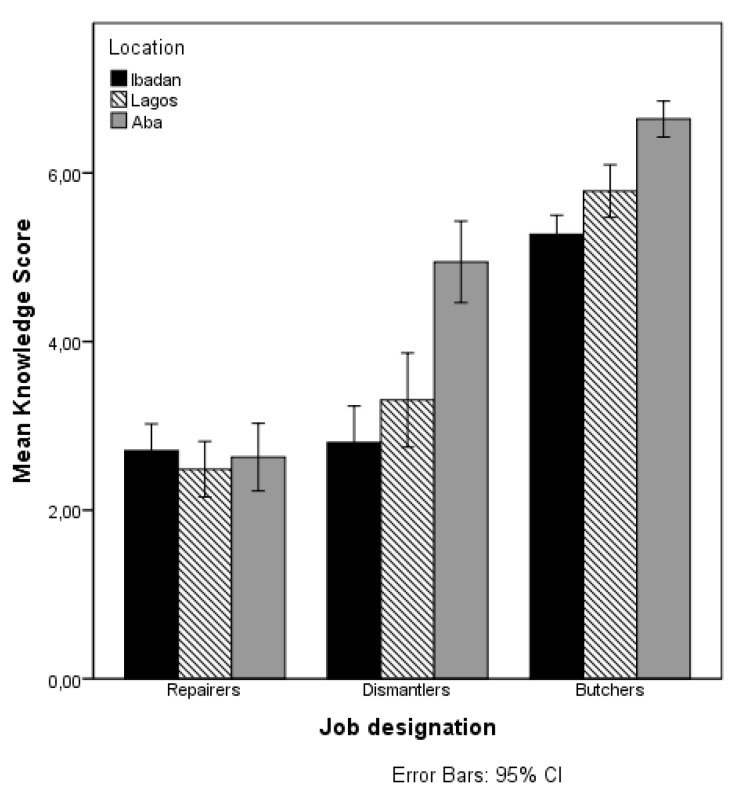
Mean knowledge score by job designation and location.

**Figure 4:**
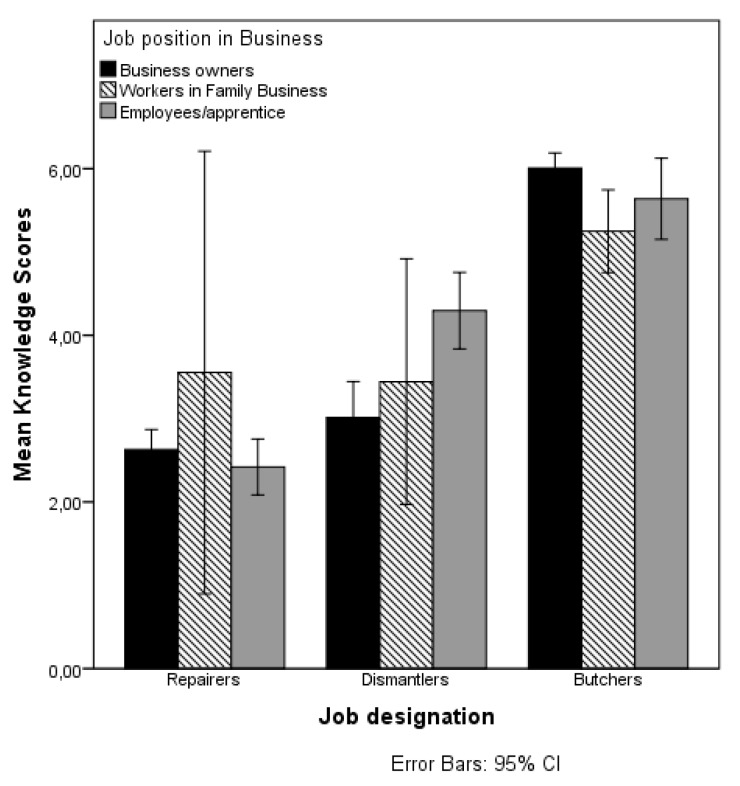
Mean knowledge score by job designation and job position in business.

**Figure 5:**
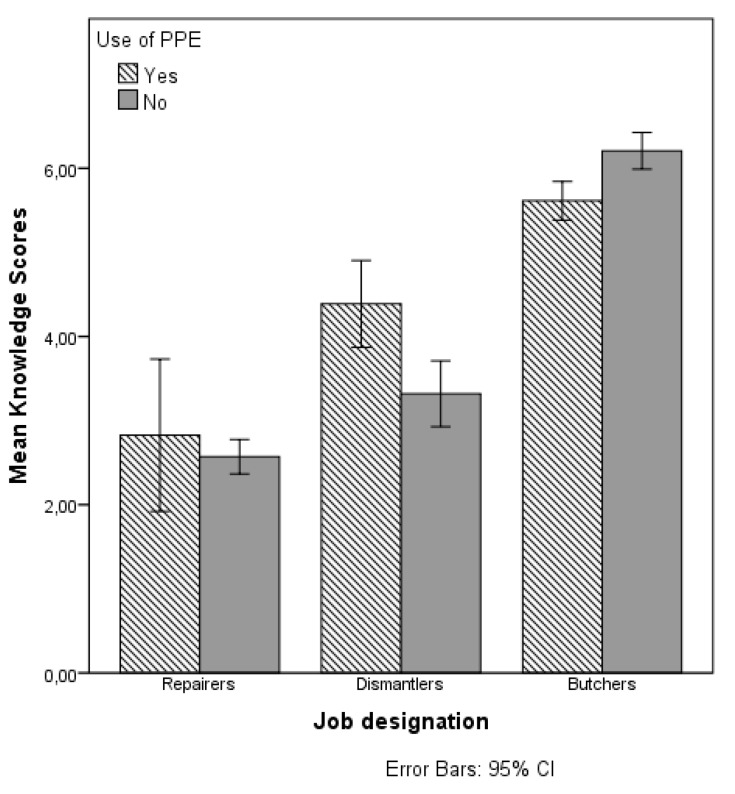
Mean knowledge score by job designation and use of PPE.

Table 3 presents the results of the main effects of job designation, the other explanatory variables (factor-i), and the interaction effects between the job designations and the other explanatory variables on the knowledge score. A visual depiction of the significant interaction effects of job designation and predicted variables on the mean score of knowledge are presented in Figure 3, Figure 4 and Figure 5. 

As Figure 3 shows, the interaction effect between job designation and location on knowledge score, the interaction effects may be explained from the high knowledge score of dismantlers in Aba. Figure 4 shows that the effect of job designation on the knowledge also depends on the worker’s position in business. Particularly for dismantlers there is a large difference in the knowledge score of the employees/apprentices compared to the business owners. Among the repairers and butchers, there is no difference in the mean knowledge scores of the employees/apprentices compared to the business owners. There is clearly more variability in the mean knowledge scores of people working in family businesses. As Figure 5 shows, the use of PPE is particularly related to higher knowledge scores for dismantlers, but not for the other two job designations. 

### 3.4. Assessment of Attitude towards Health and Safety at Work

**Figure 6:**
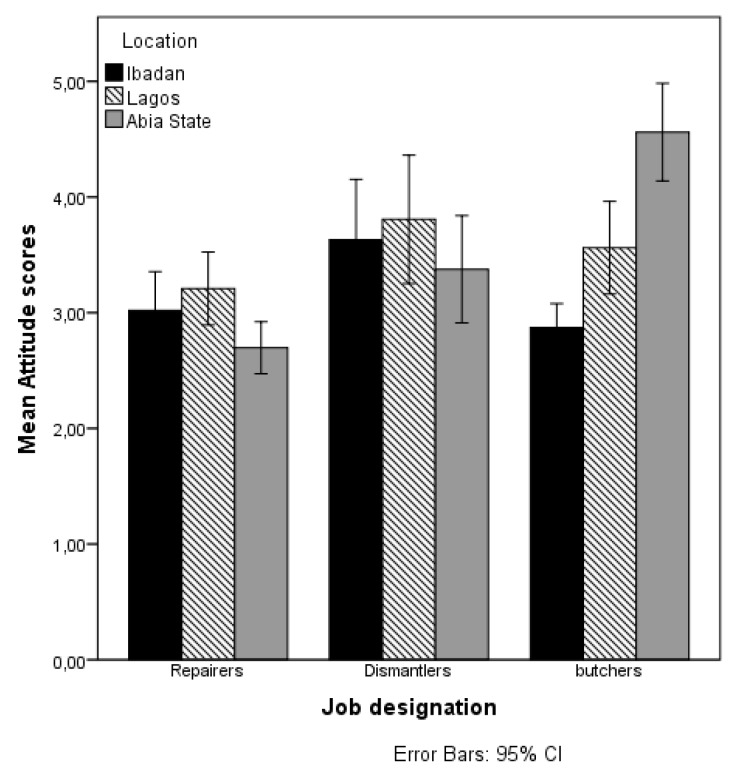
Mean attitude scores by job designation and location.

The workers’ opinions reflect their attitudes towards their health as a result of their job. About 90% of the e-waste workers (95% repairers, 84% dismantlers) and 84% of the butchers did not perceive injury at work serious enough to worry about. Only about 17% of e-waste workers (14% repairers, 19% dismantlers) and 18% of the butchers believed that the sicknesses they suffered in the last 12 months could be as a result of their jobs. About 70% of the workers did not worry about their health as a result of their jobs, and the majority (86% repairers, 88% dismantlers, 86% butchers) of them felt very good about their health. Despite the dangerous work conditions, only about 4% of the e-waste workers (7% repairers, 10% dismantlers) and 23% of the butchers mentioned worrying about their health. Regarding their major concerns, the majority (51%) of e-waste workers (54% repairers, 47% dismantlers) and 48% of the butchers reported that their major concern is finance (making more money); only 4% of the repairers, 3% of the dismantlers, and 23% of the butchers mentioned health as their major concern. The mean attitude scores across the three job designations were significantly different (F (2497) = 8.878, *p* = 0.000, ŋ^2^ = 0.034; explaining 3.4% of the variation). The mean attitude scores of the participants were: repairers 3.0 ± 1.1, dismantlers 3.6 ± 1.7, and butchers 3.7 ± 1.7; reflecting that the butchers had a better attitude towards their health in relation to their jobs (see Table 1 and Table 2). Factors that influence the attitude scores of the workers were determined by following the steps explained in Section 3.3. Table 4 presents the results of the main effects of job designation, the other explanatory variables (factor-i), and the interaction effects between the job designations and the other explanatory variables on the attitude score. A visual depiction of the significant interaction effects of job designation and predicted variables on the mean score of attitude is presented in Figure 6.

### 3.5. Assessment of Work Practices

**Figure 7:**
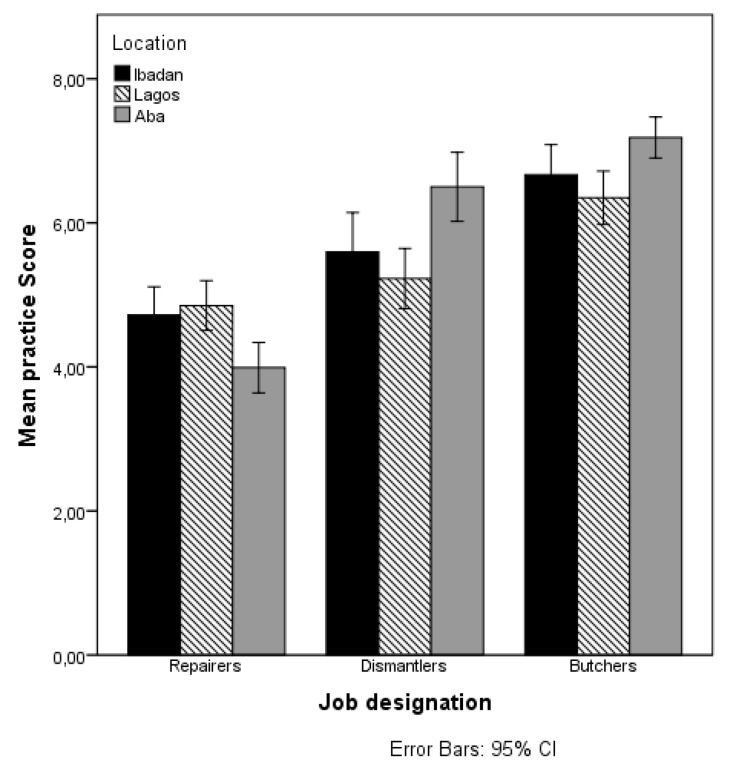
Mean Practice scores by Job designation and Location.

**Figure 8:**
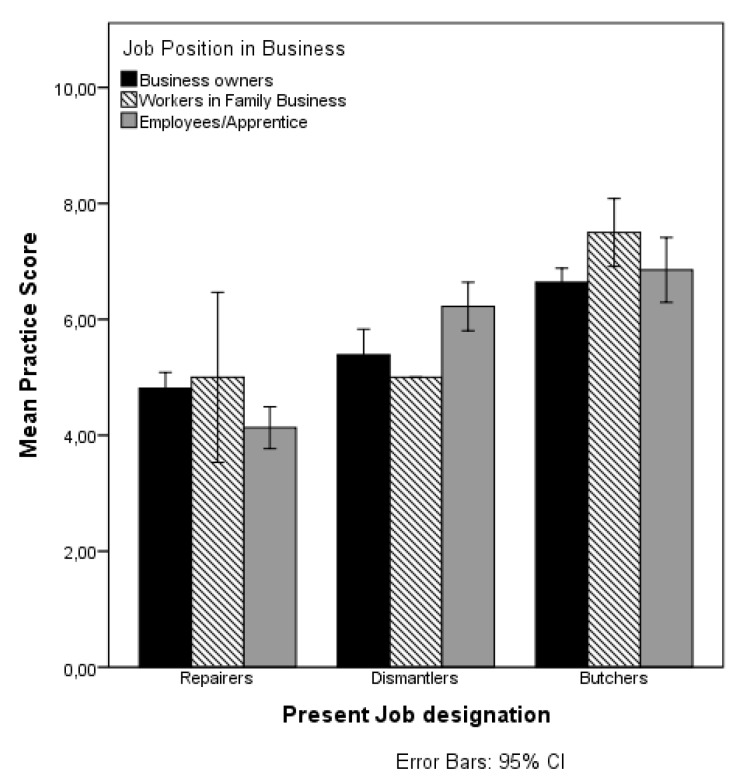
Mean Practice scores by Job designation and Job Position in Business.

Only 18% of the e-waste workers (7% repairers, 32% dismantlers) and 55% of the butchers used at least one type of PPE either most of the time or occasionally. The types of PPE commonly used by e-waste workers were gloves (13%), nose masks (7%), and boots (8%). Butchers used gloves (10%), nose masks (8%), boots (12%), overalls (20%), and head covers (15%). None of the workers used safety glasses, ear plugs or helmets. About 45% of the e-waste workers (repairers 49%, dismantlers 40%) and 60% of the butchers took their work clothing or shoes home for laundering. The majority (68.5%) of the e-waste workers (63% repairers, 75% dismantlers) and 74% of the butchers washed their hands before eating while at work. Many workers (66% e-waste workers, 42% butchers) carried heavy loads at work and the majority (55% e-waste workers, 73.3% butchers) reported uncomfortable (bent/twisted upper body) work position at work. The mean practice scores across the three job designations were significantly different (F (2497) = 88.261, *p* = 0.000, ŋ^2^ = 0.262); explaining 26.2% of the variation. The mean practice scores of the participants were: repairers 4.6 ± 1.35, dismantlers 5.8 ± 1.63, and butchers 6.73 ± 1.6. This implies that the butchers had a safer work practice compared to the e-waste workers (Table 1 and Table 2). Overall, only 32% of the e-waste workers (repairers 16%, dismantlers 50%) compared to 70% of the butchers had a total score of >5 and were categorized as having a safe practice. Factors that influence the practice scores of the workers were determined by following the steps explained in Section 3.3. Table 5 presents the results of the main effects of job designation, the other explanatory variables (factor-i), and the interaction effects between the job designations and the other explanatory variables on the mean practice scores. A visual depiction of the significant interaction effects of job designation and predicted variables on the mean practice scores are presented in Figure 7 and Figure 8.

## 4. Discussion

As far as we are aware, this is the first study that conducted detailed analyses of the Knowledge, Attitude, and Practice (KAP) of e-waste workers in the informal sector in Nigeria. Our study population showed overall poor knowledge of occupational health risks among e-waste workers compared with butchers. Overall, the occupational health risk awareness level is dependent on job designation, location and position in the business, especially among dismantlers. The strengths of this study are its focus on the informal e-waste recycling sector which is not commonly studied, assessment of the occupational health risk awareness level (knowledge, attitudes and practices) of the workers, comparison of the health risk awareness level of e-waste workers with a control group (butchers) of the same socio-economic status, use of the same research tools (adapted for butchers) to access the different work groups, large sample size, distribution of respondents across three major cities in two different geopolitical zones in Nigeria, and the use of both closed and open ended questions for the KAP assessment. However, the accuracy of the data is dependent on the ability of the respondents to be open to give the correct response irrespective of fear of government actions as a result of their responses.

The three job designations had significantly different mean knowledge, attitude, and practice scores; showing that the groups are different from each other and are from different populations. Despite the international awareness of the negative impacts of e-waste activities on the environment and human health, e-waste workers showed overall poorer knowledge (88%), more negative attitude (74%), and more unsafe practices (58%) compared to butchers on the potential health risks inherent in their jobs. However, none of the groups had remarkable knowledge, attitude or practice. This confirms the statements made by Widmer et al. [30], Borthakur [20] and Lundgren [2] who claimed that most of the participants in the informal e-waste recycling sector are not aware of environmental and health risks and do not know of better practices. Although the repairers were better educated than the dismantlers, the dismantlers were significantly more aware of the likelihood of health risks associated with their jobs. This could be due to the obviously risky work practices, and unhygienic work environment in which they work, which is contrary to the work environment of repairers although some of the repairers work with printer and photocopier toners which are considered dangerous [31]. Similarly, the general public is unaware of the dangers inherent in informal e-waste recycling. The majority of the people in India [20,32,33] and in Onitsha, Nigeria [34] were not aware of the harmful content of e-waste. In contrast, a higher proportion of informal workers in other fields, such as municipal solid waste workers (61%) in Ethiopia [35], and 63% of the butchers in Ibadan Nigeria [36] were reported to be more aware of the health risk inherent in their jobs, although awareness did not translate to good practice. However, in Aba, Nigeria, 82% of the scavengers are not aware of the dangers inherent to their jobs [37].

Regarding likely health problems as a result of their jobs, the majority (77%) of e-waste workers did not think that their jobs pose any health problem. These workers stated that there are many factors that can cause illness. Furthermore, only 3.5% of the e-waste workers and 23% of the butchers were concerned about their health as a result of their jobs. This shows that most workers in the informal sector work mainly for economic benefits. This is in agreement with the results of a study in Aba by Nzeadibe et al. which stated that 82% of the scavengers do not believe that there are health problems related to their jobs [37].

The significant effects of the location on the mean scores could be caused by the fact that each geographic location is dominated by one major ethnic group (this is highly correlated with location) which in turn influences their attitude/believes and practices. The location effect suggests that any health intervention should consider ethnicity and location. Furthermore, most the dismantlers and repairers in our study are from two different regions of the country respectively. This is confirmed by studies which stated that most of the dismantlers in Ghana [38] and Nigeria [25] are from a particular region of the country. In our study, employees and apprentices seem to have better knowledge and practices than their employers or business owners, most likely because the employees are more involved in doing the actual work and are newer in the business; they are more likely to identify health changes they experience in their new pursuit, while the business owners handle other aspects of the businesses and are no longer aware of health changes they probably experienced when they started the business. Surprisingly, there was no significant interaction effect of job designation and education on the knowledge scores, but there were interaction effects of job designation and education on attitude and practice scores. This agrees with the study by Asampong et al. (2015) [38], that states that lack of formal education of e-waste workers in Ghana influenced their attitude and practices. 

When considering the results reported here, it is likely that the results obtained are affected by the nature of the responses given by the respondents. First of all, the need for awareness as reported by dismantlers seemed to have suddenly dawned on them during the interview, whereby the interview seems to have acted as a sudden wake-up call for most of these workers; they indeed confessed that this was the first time they were being interviewed about their jobs in relation to their health Some of the workers were also in doubt whether their responses would be used against them. This doubt is induced by the fact that in recent times, the Nigerian government has put in place stricter regulations on importation of used electronics [39]. Given that these effects may have overestimated the awareness of dismantlers particularly, it re-emphasizes the low awareness levels of people working in the informal e-waste sector, e.g. compared to butchers.

The workers’ knowledge, attitude, and practice levels is dependent on the interaction effects between job designation and the other explanatory variables (location/ethnicity, position in business and use of Personal Protective Equipment (PPE) showing that these factors are very much intertwined. Therefore to plan for any health awareness/education programme for the e-waste workers or other workers in the informal sector, these factors should be considered. We anticipate that this work will help decision makers to understand the informal setting, this group of workers, and also to contribute to change in redesigning more effective e-waste management strategies/plans considering the realities of the e-waste workers in Nigeria.

### Social-Cultural Context of the Research Setting

The workers in the informal sector/economy form informal associations/networks, which are usually formed by a group of people who are informally bound together by mutual needs or common interest, and membership is voluntary. They establish their own leadership, set rules that govern them, and they meet regularly. These associations usually provide them with emotional and financial support, as well as sharing of knowledge. These associations also give them a sense of belonging [40,41]. The strength of informal associations is that members feel the need to belong to the association, therefore the associations are sustained. In Nigeria informal associations are usually formed along ethnic lines and common business interest. These associations have proved to operate efficiently without any formal support. However, they often work in cross-purposes with formal sector/economy [40]. The formal sector, international organizations and academics do not understand the dynamics of African informal economy, so they tend to represent it in terms of criminality or cultural dysfunction, therefore they treat the informal sector as structurally inadequate and irrelevant [40]. 

It is important to note that occupational exposure to health risk and accidents occur in both formal and informal settings, and those occurrences could be due to human error [42,43], lack of risk assessment and risk management. However in formal and complex settings, there are organizational occupational safety and health (OS&H) programmes and policies put in place to minimize exposure to health risks in work places but in the informal settings, OS&H are nonexistent, mainly because during the formulation of the policies and regulations, the informal sector is not considered or involved, which leads to difficulties in implementation of government policies and regulations. To improve OS&H in the informal sector, formal institutions such as the National Environmental Standards and Regulations Enforcement Agency (NESREA) should appreciate, understand and work with the informal associations. The enforcement agencies must be seen not to be antagonistic so as to influence the workers in the informal sector to align with government policies. Although the informal associations are self-organizing, they can be encouraged to abide by government regulations, if they perceive that they are appreciated by the enforcement agencies. NESREA in combination with Ministry of Health can work together with the informal associations to develop effective grassroot communication methods which will improve the workers’ health risk knowledge and work practices.

## 5. Conclusions

Informal workers often underestimate the health risks associated with their jobs. It is important that workers in the informal settings are aware of the potential health risks peculiar to their jobs and the safety measures to be undertaken. One way to improve the awareness level is for formal institutions to work with the informal associations to communicate the health risks and OS&H associated with their jobs. Our findings provide a scientific basis crucial to devising appropriate OS&H intervention for e-waste workers and other workers in the informal sector. Health risk awareness level of the e-waste workers were significantly lower compared with their counterparts (butchers) in the same informal sector. Since the level of knowledge correlated with practice, increasing the workers’ health risk knowledge may decrease risky work practices; suggesting that a bottom-up approach in reducing health risk practices in the informal sector may be more effective. However, to implement health risk awareness programmes in the informal sector, it should be borne in mind that the approach may differ depending on the type of job performed, location, and workers’ position in the business.

## Figures and Tables

**Table 1:** Knowledge, attitude, and practice mean scores by job designation, location, job position, use of Personal Protective Equipment (PPE), and education.

Predicted Variables		Knowledge Score	Attitude Score	Practice Score
		Mean ± SD	Mean ± SD	
Job designation	Repairers	2.6 ± 1.2	3.02 ± 1.13	4.6 ± 1.35
	Dismantlers	3.7 ± 1.8	3.6 ± 1.66	5.8 ± 1.63
	Butchers	5.9 ± 1.2	3.67 ± 1.68	6.73 ± 1.6
Location	Ibadan	3.9 ± 1.7	3.11 ± 1.24	5.8 ± 1.9
	Lagos	4 ± 2.1	3.5 ± 1.61	5.5 ± 1.6
	Aba	5.2 ± 2.0	3.78 ± 1.70	6.2 ± 1.8
Job position in business	Business owners	2.6 ± 1.2	3.53 ± 1.58	5.9 ± 1.8
	Workers in family business	3.5 ± 2.1	3.56 ± 1.66	6.4 ± 1.6
	Employees/apprentices	2.4 ± 1.2	3.25 ± 1.43	5.6 ± 1.8
Use of PPE	No	3.92 ± 2.11	3.4 ± 1.51	5 ± 1.4
	Yes	5.13 ± 1.56	3.57 ± 1.64	7.5 ± 1.2
Education	No formal education	5.2 ± 1.8	3.5 ± 1.5	6.5 ± 1.9
	Primary	5.2 ± 1.8	3.66 ± 1.78	6.5 ± 1.7
	Secondary	3.9 ± 2.0	3.36 ± 1.42	5.5 ± 1.7
	Post-Secondary	2.9 ± 1.5	3.37 ± 1.66	5.3 ± 1.2

**Table 2:** One-Way ANOVA output for the Knowledge, Attitude, and Practice scores on a 1–10 point scale.

Variable	Knowledge Score	Attitude Score	Practice Score
Job designation	F (2497) = 269.582	F (2497) = 8.878	F (2497) = 88.261
	*p* = 0.000	*p* = 0.000	*p* = 0.000
	ŋ^2^ = 0.520	ŋ^2^ = 0.034	ŋ^2^ = 0.262
State/Location	F (2497) = 21.928	F (2497) = 7.715	F (2497) = 6.045
	*p* = 0.000	*p* = 0.001	*p* = 0.003
	ŋ^2^ = 0.081	ŋ^2^ = 0.030	ŋ^2^ = 0.024
Migration status	F (1498) = 15.277	F (1498) = 0.054	F (1498) = 12.822
	*p* = 0.000	*p* = 0.817	*p* = 0.000
	ŋ^2^ = 0.030	ŋ^2^ = 0.000	ŋ^2^ = 0.025
Education	F (3496) = 26.886	F (3496) = 1.125	F (3496) = 15.678
	*p* = 0.000	*p* = 0.338	*p* = 0.000
	ŋ^2^ = 0.140	ŋ^2^ = 0.007	ŋ^2^ = 0.087
Position in Business	F (2497) = 4.039	F (2497) = 1.688	F (2497) = 2.965
	*p* = 0.010	*p* = 0.186	*p* = 0.052
	ŋ^2^ = 0.018	ŋ^2^ = 0.007	ŋ^2^ = 0.012
Use of PPE	F (1498) = 143.406	F (1498) = 1.350	F (1498) = 377.618
	*p* = 0.000	*p* = 0.246	*p* = 0.000
	ŋ^2^ = 0.080	ŋ^2^ = 0.003	ŋ^2^ = 0.431
Age (continuous)	F (1498) = 52.459	F (1498) = 0.257	F (1498) = 16.523
	*p* = 0.000	*p* = 0.612	*p* = 0.000
	R = 0.309	R = 0.023	R = 0.179
Years of work experience (continuous)	F (1498) = 6.937	F (1498) = 0.003	F (1498) = 0.829
	*p* = 0.009	*p* = 0.960	*p* = 0.363
	ŋ^2^ = R = 0.117	ŋ^2^ = R = 0.002	ŋ^2^ = R = 0.041

Partial Eta squared (ŋ^2^) = Effect size.

**Table 3:** *F*-values of the Two-Way ANOVA for the knowledge score.

Factor-i	Job Designation (ŋ^2^)	Factor-i (ŋ^2^)	Job # Factor-i (ŋ^2^)
State/Location	312.474 (0.560) ***	31.724 (0.114) ***	8.605 (0.066) ***
Migration status	195.293 (0.442) ***	0.000 (0.000)	0.375 (0.002)
Education	46.839 (0.161) ***	0.968 (0.006)	1.043 (0.011)
Job Position	73.802 (0.231) ***	1.344 (0.005)	7.835 (0.060) ***
Use of PPE	144.044 (0.368) ***	1.854 (0.004)	13.626 (0.052) ***
Age (ANCOVA)	20.421 (0.076) ***	1.082 (0.002)	0.248 (0.001)
Years of work experience (ANCOVA)	109.069 (0.306) ***	1.34 5 (0.003)	1.761 (0.007)

*** *p* < 0.001, Partial Eta Squared = ŋ^2^, Interaction = #.

**Table 4:** *F*-values of the Two-Way ANOVA for the Attitude score.

Factor-i	Job Designation (ŋ^2^)	Factor-i (ŋ^2^)	Job # Factor-i (ŋ^2^)
State/Location	10.789 (0.042) ***	3.165 (0.013) *	9.721 (0.073) ***
Migration status	10.202 (0.040) ***	0.199 (0.000)	1.284 (0.005)
Education	4.860 (0.019) **	2.543 (0.015)	3.025 (0.030) **
Job Position	1.363 (0.006)	1.213 (0.005)	0.217 (0.002)
Use of PPE	3.334 (0.013) **	0.355 (0.001)	0.655 (0.003)
Age (ANCOVA)	0.779 (0.003)	0.165 (0.000)	0.028 (0.000)
Years of work experience (ANCOVA)	6.135 (0.024) **	0.016 (0.000)	0.974 (0.004)

*** *p* < 0.001, ** *p* < 0.01,* *p* < 0.05, Partial Eta Squared = ŋ^2^, Interaction = #.

**Table 5:** *F*-values of the Two-Way ANOVA for the practice score.

Factor-i	Job designation (ŋ^2^)	Factor-i (ŋ^2^)	Job # Factor-i (ŋ^2^)
State/Location	312.474 (0.560) ***	31.724 (0.114) ***	8.605 (0.066) ***
Migration status	195.293 (0.442) ***	0.000 (0.000)	0.375 (0.002)
Education	46.839 (0.161) ***	0.968 (0.006)	1.043 (0.011)
Job Position	73.802 (0.231) ***	1.344 (0.005)	7.835 (0.060) ***
Use of PPE	144.044 (0.368) ***	1.854 (0.004)	13.626 (0.052) ***
Age (ANCOVA)	20.421 (0.076) ***	1.082 (0.002)	0.248 (0.001)
Years of work experience (ANCOVA)	109.069 (0.306) ***	1.345 (0.003)	1.761 (0.007)

*** *p* < 0.001, Partial Eta Squared = ŋ^2^, Interaction = #.

## Supplementary Materials

The following are available online at www.mdpi.com/link/1660-4601/14/8/911/s1, Table S1: Socio-demographic characteristics of all the informal workers, Table S2: Knowledge of work health risk, Table S3: Healthy work Attitude at work, Table S4: Healthy work Practice.
