# Supplementary material for: Health Risks Awareness of Electronic Waste Workers in the Informal Sector in Nigeria
Source: Int J Environ Res Public Health. 2017 Aug 13;14(8):911. doi: 10.3390/ijerph14080911 (PMC5580614; doi:10.3390/ijerph14080911)
Supplement: Supplementary file 1 [file ijerph-14-00911-s001.pdf]

Supplementary Materials: Health Risks Awareness of Electronic Waste Workers in the Informal Sector in Nigeria

Chimere M. Ohajinwa, Peter M. Van Bodegom, Martina G. Vijver and Willie J. G. M. Peijnenburg

Table S1. Socio-demographic characteristics of all the informal workers.

Demographic characteristics		Job Designation		
		**Repairers = 153 n(%)**	**Dismantlers = 126 n(%)**	**Butchers = 221**
Location	Lagos	67 (43.8)	44 (34.9)	73 (33)
	Ibadan	48 (31.4)	42 (33.3)	74(33.5)
	Aba	38 (24.8)	40 (31.7)	74(33.5)
Age	30 years or less	79 (51.6)	81 (64.3)	55(25)
	More than 30 years	74 (48.4)	45 (35.7)	166(75)
Education	No formal Education	0(0)	12 (9.5)	40(18)
	Primary	8 (5.2)	23 (18.3)	97(44)
	Secondary	105 (68.6)	85 (67.5)	79(36)
	Post-secondary	40 (26.1)	6 (4.8)	5(2)
Training	Yes	125 (81.7)	101 (80.2)	157(71)
	No	28 (18.3)	25 (19.8)	64(29)
Training type received	On-the-job training	148 (96.7)	126 (100)	219(99.1)
	Training by an expert	5 (3.3)	0 (0)	2(0.9)
Kind of employment	Permanent	136 (88.9)	112 (88.9)	206(93.2)
	Temporary	17 (11.1)	14 (11.1)	15(6.8)
Position in business	Business owner	98 (64.1)	56 (44.4)	176(79.6)
	Employee/Apprentice	50 (32.7)	60 (47.6)	27(12.2)
	Family business	5 (3.3)	10 (7.9)	18(8.1)
Years of work experience	1-10 years	115 (75.2)	101 (80.2)	96(43.4)
	More than 10 years	38 (24.8)	25 (19.8)	125(56.6)
Work-hours/ day	8 hours or less	48 (31.4)	26 (20.6)	93(42)
	More than 8 hours	105 (68.6)	100 (79.4)	128(58)
Income	N2000 or less	86 (30.8)	84 (30.1)	116(52.5)
	N2001-N5000	36 (12.9)	27 (9.7)	82(37.1)
	More than N5000	31 (11.1)	15 (5.4)	23(10.4)
Use of PPE	Yes	11(7.2)	40 (31.7)	115(52)
	No	142 (92.8)	86 (68.3)	106(48)
Ethnicity	Igbo	73 (47.7)	48 (38.1)	60 (27.1)
	Yoruba	64 (41.8)	47 (37.3)	113 (51.1)
	Hausa	0 (0)	18 (14.3)	29 (13.1)
	Others	16 (10.5)	13 (10.3)	19(8.6)

Table S2. Knowledge of work health risk.

Knowledge of Health Risk (Scores)	Job Designation		
	Repairers (153)	Dismantlers (126)	Butchers = 221(100) n(%)
For EW: what type of chemicals come from this second hand electronics during recycling<br><br>For B: what type of substances come from the animals (2)	7 (4.6)	27(21.4)	221 (100)
For EW: Do you think chemicals from second-hand electronics can affect health?<br><br>For B:Do you think substances from the animals can affect health? (1)	44 (28.8)	40 (31.7)	49 (22.2)
Which route of exposure (entrance to the body) do you think the chemicals/substances can enter your body? (1)	56 (36.6)	63 (50)	130 (58.8)
Could you list the Personal Protective Equipment (PPE) you need for your work? (2)	52 (34)	67(53.2)	154 (69.7)
What health problems or illnesses do you think can happen due to work environment? (2)	28 (18.3)	32 (25.4)	99 (44.8)
Knowledge level			
Poor Knowledge	149 (97.4)	96 (76.2)	54 (24.4)
Good knowledge	4 (2.6)	30 (23.8)	167 (75.6)
Knowledge mean score	2.59 ± 1.24	3.66 ± 1.82	5.9 ± 1.23

EW = E-waste worker, B = Butchers

Table S3. Healthy work attitude at work.

Attitude towards Health and Work(Scores)	Repairers (153) n(%)	Dismantlers (126) n(%)	Butchers = 221(100) n(%)
How would you perceive the injury you experience on this job most times (1)			
Serious	7 (4.6)	20 (15.9)	36 (16.3)
Mild/moderate	146 (95.4)	106 (84.1)	185 (83.7)
Do you think the sicknesses you had the last 12 months due to your jobs? (1)			
Yes	22 (14.4)	24 (19)	39 (17.6)
No	75 (49)	90 (71.4)	123 (55.7)
I don't know	56 (36.6)	12 (9.5)	59 (26.7)
Do you worry about your health or that of your family due to your work environment? (1)			
No, do not worry	116 (75.8)	80 (63.5)	146 (66.1)
Previously worried, but no longer worried	18 (11.8)	28 (22.2)	59 (26.7)
Little worried	9 (5.9)	6 (4.8)	8 (3.6)
Yes, very worried	10 (6.5)	12 (9.5)	8 (3.6)
What are the major challenges in your business (1 for heath)			
Health	6 (3.9)	4 (3.2)	51 (23.1)
Work environment	28 (18.3)	28 (22.2)	31 (14)
Finances	83 (54.2)	59 (46.8)	106 (48)
No response	35 (22.9)	27 (21.4)	41 (18.6)
No challenges	1 (0.7)	0 (0)	4(1.8)
Attitude score	3.02 ± 1.13	3.6±1.66	3.67±1.68
Poor Attitude (≤5)	124 (81)	81 (64.3)	139 (62.9)
Good Attitude (>5)	29 (19)	45 (35.7)	82 (37.1)

Table S4. Healthy work practice.

	Repairers (153) n(%)	Dismantlers(126) n(%)	Butchers (221) n(%)
Use of PPE			
(1) Yes	11 (7.2)	40 (31.7)	122 (55.2)
(0) No	142 (92.8)	86 (68.3)	99 (44.8)
Do you always wash your hand before eating anything while at work?			
Yes	97 (63.4)	94 (74.6)	163 (73.8)
No	56 (36.6)	32 (25.4)	58 (26.2)
Do you change your clothes after work before going home?			
Yes	14 (9.2)	65 (51.6)	170 (76.9)
No	134 (87.6)	54 (42.9)	44 (19.9)
Sometimes	5 (3.3)	7 (5.6)	7 (3.2)
How often do you bend and/twist your upper body (uncomfortable work position)			
Always/sometimes	66 (43.1)	87 (69)	148 (67)
(0) Rarely	59 (38.6)	30 (23.8)	34 (15.4)
(0) Never	28 (18.3)	9 (7.1)	39 (17.6)
Do you take your work clothes or shoes home for laundering			
Yes	75 (49)	50 (39.7)	133 (60.2)
No	72 (47.1)	75 (59.5)	88 (39.8)
Not applicable	6 (3.9)	1 (0.8)	-
Work conditions/position			
Uncomfortable work conditions	49 (32)	60 (47.6)	120 (54.3)
Take a position that is comfortable	98 (64.1)	59 (46.8)	79 (35.7)
No Response	6 (3.9)	7 (5.6)	22 (10)
Practice score mean	4.6 ± 1.35	5.8 ± 1.63	6.73 ± 1.6
Practice rate - Poor practice (≤5)	128 (83.7)	63 (50)	67 (30.3)
Good practice (>5)	25 (16.3)	63 (50)	154 (69.7)

© 2017 by the authors; licensee MDPI, Basel, Switzerland. This article is an open access article distributed under the terms and conditions of the Creative Commons by Attribution (CC-BY) license (http://creativecommons.org/licenses/by/4.0/).
